# Single-Stage Bilateral Reverse Shoulder Arthroplasty for a Bilateral Four-Part Fracture Dislocation of the Proximal Humerus in an Elderly Patient: A Case Report

**DOI:** 10.7759/cureus.49002

**Published:** 2023-11-18

**Authors:** Razan A Almaghrabi, Ali M Almousa, Abdulmalek Almulla, Omar Salem, Latifah Almana

**Affiliations:** 1 College of Medicine, Imam Abdulrahman Bin Faisal University, Dammam, SAU; 2 Orthopedic Surgery, King Fahad Specialist Hospital, Dammam, SAU

**Keywords:** dash score, constant murley score, neer's classification, reverse total shoulder arthroplasty, single stage, proximal humerus fracture

## Abstract

Proximal humerus fractures (PHFs) are a common type of fracture in adults. Although PHFs are common, bilateral presentation is extremely rare. Most PHFs are treated conservatively. In this report, we describe a 69-year-old right-hand-dominant male patient who was involved in a high-impact motor vehicle accident (MVA). The patient’s upper limbs were in a fully extended position while he was holding the driving wheel, where he sustained a side impaction to the car by a hard object that caused bilateral four-part PHF with dislocation, which was confirmed on radiological investigations. The orthopedic surgery team believed that surgical treatment was necessary and ideal for these bilateral fracture dislocations, specifically bilateral reverse total shoulder arthroplasty (RTSA). This is due to multiple factors, including the risk of humeral head avascular necrosis (AVN), the patient’s advanced age, low demand, poor bone stock, osteoporosis, and a non-fixable fracture pattern. The patient underwent a single-stage bilateral RTSA procedure, which was well tolerated. He was optimized postoperatively. The post-operative X-ray showed good and satisfactory implant positions and orientation. Functional assessment using the Constant-Murley Score (CMS) and Disabilities of the Arm, Shoulder and Hand (DASH) score were calculated at three-months follow-up (right-left: 50-60 and 41-14, respectively), at five-months follow-up (right-left: 34-66 and 38-14, respectively), and at eight-months follow-up (right-left: 40-68 and 24-7.5, respectively). Follow-up X-rays revealed good tuberosities healing, and no loosening or scapular notching. In addition, pain was assessed on a numerical rating scale (NRS), which demonstrated fast pain relief. Short-term follow-up with the patient demonstrated that he was satisfied with the surgery, especially the left side with a pain score on the NRS of one. We selected to share our experience of this complex case with our peers in the field of orthopedic surgery worldwide so that such a procedure could be implemented in similar cases to ensure satisfactory outcomes following bilateral four-part PHF with dislocation.

## Introduction

Proximal humerus fractures (PHFs) are the seventh most common type of fractures in adults. According to several studies conducted in various populations, their prevalence ranges from 4% to 10% of all fractures [1]. PHFs are most common in patients over the age of 65 years, accounting for 10% of fractures in this age group. Of all osteoporotic fractures, PHFs are considered the third most common type with a 13% lifetime risk for women aged 50 years and above. Such an injury represents a bimodal incidence; it typically occurs in young individuals as a result of high-energy trauma and in elderly people as a result of low-energy falls [2]. Launonen et al., 2015, reported the total incidence of PHFs as 82 per 100,000 persons per year [3].

The Neer classification assorts PHFs into four categories: one-part, two-part, three-part, and four-part fractures [4]. According to this classification, the majority of these fractures are one- or two-part fractures, whereas four-part fractures are relatively rare and account for about 3% of all PHFs, with simultaneous bilateral PHFs being uncommon. Furthermore, simultaneous bilateral four-part fractures are rare and less common. With infrequent published reports discussing treatment plans, techniques, and outcome with non-operative, open reduction and internal fixation (ORIF) or simultaneous bilateral reverse total shoulder arthroplasty (RTSA) [5-10]. Bilateral PHFs are frequently associated with dislocation [11]. The triple "E" syndrome refers to the causes of bilateral fracture dislocations (epilepsy, electrocution, and extreme trauma), with seizure episodes accounting for most of the cases [6].

PHFs could be treated operatively or non-operatively. Surgical options include minimally invasive osteosynthesis, ORIF, closed reduction and percutaneous pinning (CRPP), intramedullary nailing (IMN), hemiarthroplasty (HA), and RTSA [12,13]. Regardless of the treatment chosen for PHFs, the risk of humeral head avascular necrosis (AVN) remains. Hertel's criteria are an essential tool for predicting humeral head ischemia following intracapsular PHFs. As determined by Hertel et al. in 2004, good predictors are those that decrease the incidence of humeral head ischemia include: the length of the metaphyseal head extension, which is called calcar length attached to articular segment < 8 mm, the integrity of the medial hinge, and the basic ‘‘LEGO system’’ fracture pattern (types 2-9-10-11-12). Then they mentioned the following predictors as poor-moderate predictors that increase the incidence of humeral head ischemia which include: three- and four-fragment fracture, angular displacement of the head (>45°), the amount of displacement of the tuberosities (>10 mm), glenohumeral dislocation, and head split components. Thus, Hertel’s criteria serve as an invaluable tool for surgical planning and preoperative evaluation [14,15]. 

There are two ways to do bilateral RTSA as mentioned in the literature. The first way is called simultaneous bilateral shoulder arthroplasty, which means there are two teams working on each side at the same time. The second way is called single-stage, which means one surgeon performs the procedure on one side, and then the same surgeon operates on the contralateral side under one anesthetic. Our case counts as a single-stage bilateral RTSA. To the best of the authors’ knowledge, six case reports of single-stage or simultaneous bilateral RTSA for bilateral PHFs have been published [5-10]. Three of these cases were as a result of falls [5,6,8], and the other three were because of seizures [7,9,10]. All the reported cases demonstrated good outcomes, although long-term follow-ups were needed [5-10]. In this case report we report a case of a 69-year-old male patient, a known case of hypertension, diabetes mellitus type ll, and hypothyroidism. The patient was presented one week after the motor vehicle accident (MVA) with bilateral four-part PHF with dislocation as a referral case from another hospital. He was an unrestrained driver (not wearing a seat belt) with no history of ejection from the car. The MVA mechanism was a side impaction to the car by a hard object. He was treated with single-stage bilateral RTSA, with eight months of follow-up.

At our hospital, Institutional Review Board approval (IRB) was obtained for this case report. In addition, the patient gave his informed consent to the surgical procedure as well as the publication of the report.

## Case presentation

A 69-year-old right-hand-dominant retired male patient, a non-smoker, was involved in an MVA. The patient was referred to our hospital from another institute where he was initially managed with shoulder immobilizers, and then referred and transferred to our service via an ambulance for further management. The patient was a victim of a frontal car collision on a highway road driving at a speed of about 120 km/hr with no seat belt on. There was no ejection, head trauma, loss of consciousness, or death at the scene. He reported that his upper limbs were in a fully extended position while he was holding the driving wheel. Then he sustained a side impaction to the car by a hard object that resulted in bilateral four-part PHF with dislocation. The patient had no sensory deficits. Detailed motor examination couldn’t be done due to pain, but the patient had deltoid contraction alongside weak elbow and wrist function as a result of pain. Later on, we conducted a nerve conduction study (NCS) and electromyography (EMG) of the right side, as it was the worst affected side. The results were discussed with the neurologist and showed a lesion involving the upper trunk of the brachial plexus which was likely from the fracture dislocation on initial trauma with signs of regeneration. 

The patient's medical history was remarkable for hypertension, diabetes mellitus type ll, and hypothyroidism on medications. Upon evaluation of functional abilities and the activities of daily living (ADLs), the patient reported complete independence. Upon presentation, vital signs were stable. The patient had an average body build for a Saudi male with a BMI of 25 kg/m^2^. He was alert, oriented, conscious, and complaining of pain in both shoulders that responded to analgesia. Musculoskeletal examination revealed deformed shoulders bilaterally with intact skin and bruises over the anterolateral aspect of the right shoulder, with generalized tenderness over both shoulder joints. Range of motion (ROM) was very limited and impaired in both shoulders due to pain. It was difficult to perform a full neurological examination of the elbow and hand in detail due to pain. 

The patient was admitted under the orthopedic surgery team through the emergency department. In the emergency department, pain management was provided through arm slings and proper analgesia. Pre-operative preparation was started through obtaining full laboratory and necessary radiological investigations. Internal medicine team were consulted to clear the patient for surgery and for a pre-operative and post-operative plan of care regarding his comorbidities. 

Imaging

X-rays

**Figure 1:**
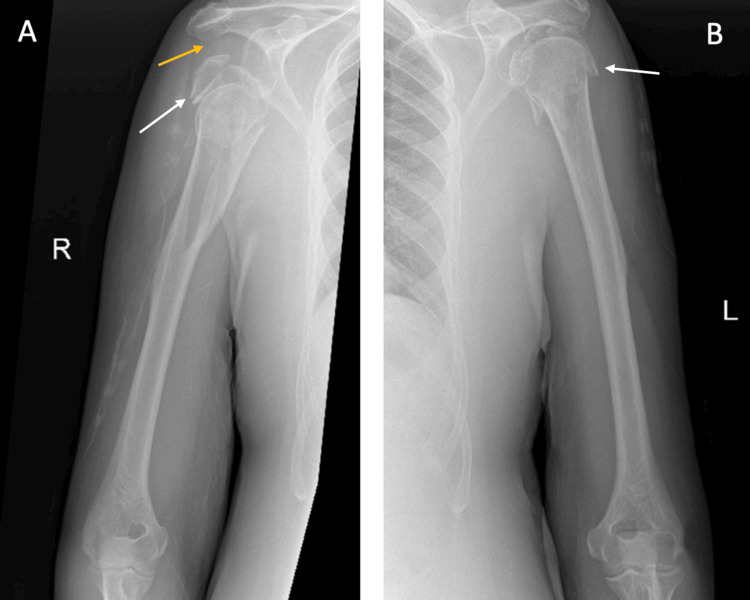
Pre-operative X-ray of the right (A) and left (B) shoulders and humeri. (A) Anteroposterior X-ray of the right shoulder including the right humeral shaft demonstrating a markedly displaced, angulated, and comminuted four-part proximal humerus fracture with significant inferior dislocation. (B) Anteroposterior X-ray of the left shoulder and left humerus showing displaced and comminuted four-part left proximal humeral head fracture with inferior subluxation of the humeral head. White arrows point to the fracture site. Yellow arrow shows the dislocation.

Anteroposterior X-ray of the left shoulder and left humerus were obtained which showed a displaced and comminuted four-part left proximal humeral head fracture with inferior subluxation of the humeral head. Also, an anteroposterior X-ray of the right shoulder including the right humeral shaft was obtained. The right side demonstrated a markedly displaced, angulated, and comminuted four-part proximal humerus fracture with significant inferior dislocation (Figure 1). 

CT Scan

**Figure 2:**
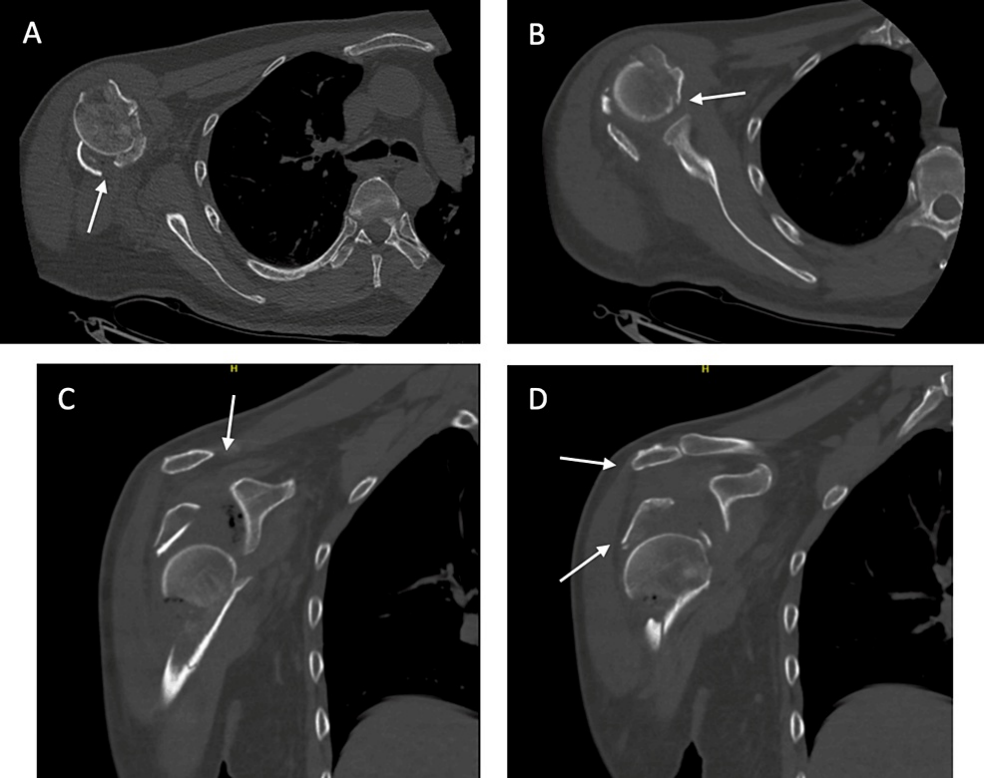
(A-D) Pre-operative CT of the right shoulder. (A, B) Right shoulder axial cut CT scan illustrating the comminution of the proximal humerus. (C, D) Right shoulder coronal cut CT scan illustrating the comminution of the proximal humerus with dislocation of the glenohumeral joint. White arrows point towards the fracture sites. CT: Computed tomography.

**Figure 3:**
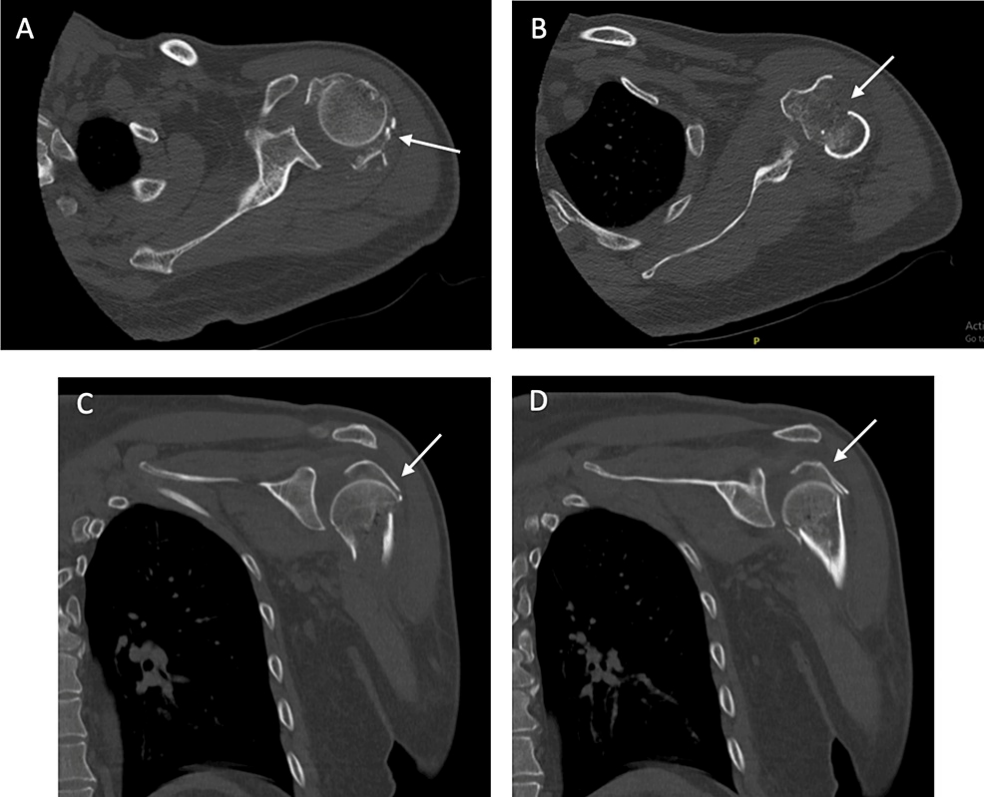
(A-D) Pre-operative CT of the left shoulder. (A, B) Left shoulder axial cut CT scan illustrating the comminution of the proximal humerus. (C, D) Left shoulder coronal cut CT scan illustrating the comminution of the proximal humerus with dislocation of the glenohumeral joint. White arrows point towards the fracture sites. CT: Computed tomography.

**Figure 4:**
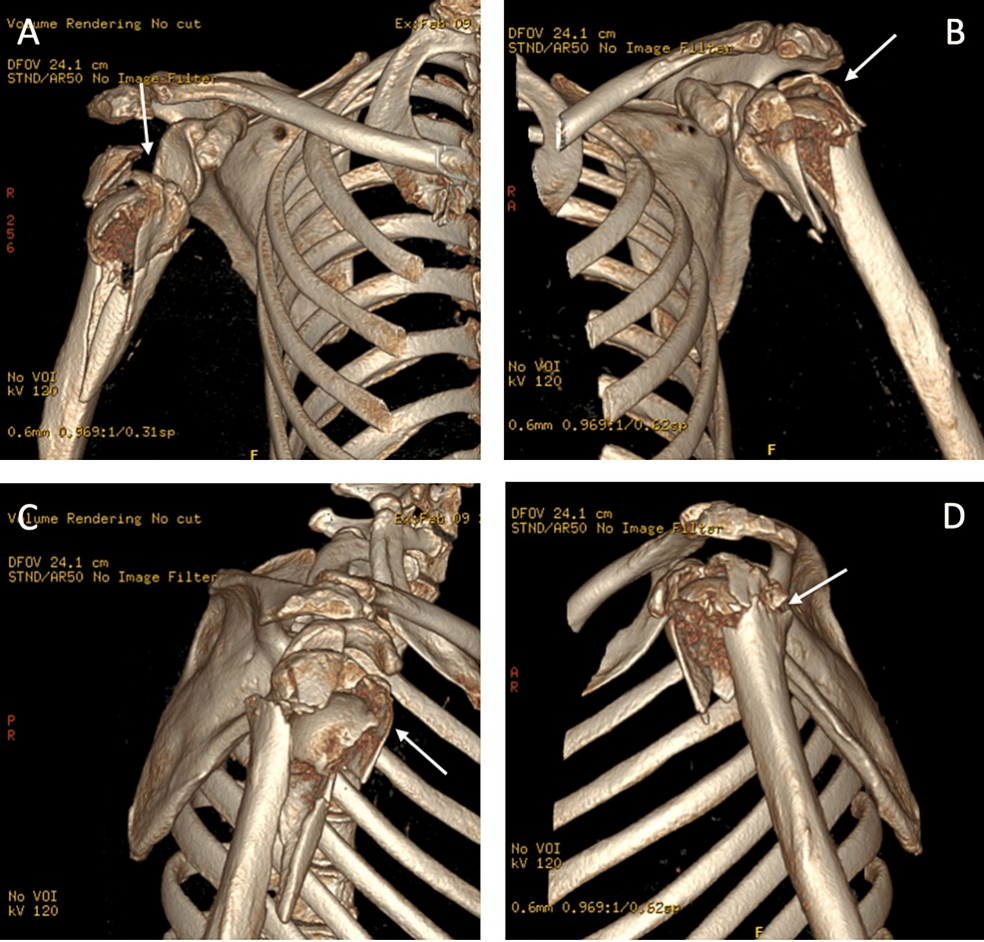
Pre-operative CT 3D reconstruction of the right (A, B) and left (C, D) shoulders demonstrating the comminution of fracture. White arrows point towards the fracture sites. CT: Computed tomography.

A bilateral shoulder joint CT (computed tomography) scan without IV contrast with three-dimensional reconstruction was performed (Figures 2-4). It re-demonstrated comminuted displaced fracture of the left and right proximal humerus involving four parts according to Neer’s classification, with inferior dislocation of the glenohumeral joints bilaterally.

Treatment planning

Treatment options were discussed with the patient in the context of his age and pattern of fracture. The orthopedic surgery team believed that surgical treatment was necessary for these bilateral fractures, specifically, single-stage bilateral RTSA. According to Hertel’s criteria [15], these predictors of humeral head AVN were met: left-side medial calcar < 8 mm, right-side angulation > 45° with calcar length < 2 mm, bilateral comminution and complex geometry of fracture, alongside bilateral medial hinge disruption. Furthermore, considering the patient’s advanced age, low demand, poor bone stock, osteoporosis, and non-fixable fracture pattern, the orthopedic surgery team decided that the patient would benefit more by receiving a prosthesis. Therefore, single-stage bilateral RTSA would result in the most predictable clinical outcome, the lowest risk of operation failure, and better recovery of the shoulder’s ROM and function.

Surgical technique

The patient provided informed consent for surgery, in addition to the publication of his case. He was operated on 11 days from the time of the injury. The team chose to start with the right shoulder since the fracture dislocation was more severe and challenging on the right side. The left shoulder was addressed in a similar manner, except for the cerclage wire which was used on the right side only. They were not prepared and draped simultaneously to ensure field sterility. Thus, after placing the dressing and shoulder immobilizer on the right shoulder, preparation of the left shoulder was started. The patient was brought into the operative theater and placed supine on the operative table. General and regional anesthesia were provided. 2 g of intravenous cefazolin was given as a prophylactic antibiotic at induction, followed by another dose mid-surgery. All bony prominences were padded, and the patient was positioned in a semi-sitting beach chair position at 30 degrees with medial support to the scapula for optimal positioning and to gain full shoulder ROM. A bilateral lower limb pneumatic compression device was applied throughout the surgery as a deep vein thrombosis (DVT) precaution. After prepping and draping, time out was done. The deltopectoral approach was utilized with a more distal extension on the right side. The long head of the biceps tendon was tenodesed to the released pectoralis major. Subscapularis and the lesser tuberosity were tagged, and the same thing was done for the greater tuberosity and the cuff tendons at the tendon-bone junction. The split humeral head was then extracted. For both shoulders, Tornier Aequalis Reversed FX shoulder prosthesis (Tornier, Grenoble, France) was used. The glenoid was prepared using the 10-degree inferior tilt guide. A size 29 base plate was fixed with a 25 mm central post on the right. Compression and locking screws were inserted into all holes of the base plate. A size 36 standard glenosphere** **was used. The humerus was then prepared and trialed. Two cerclage wires were placed in the right humeral shaft and tied to the fracture extension. The fragments were reduced and held well. After that canal preparation and cementing then insertion of humeral stem with size 9 x 210 mm. The shoulder joint was reduced and the tuberosities were repaired to each other and to the shaft and had a stable ROM. The same steps were repeated for the left shoulder without the use of a cerclage (size 29 baseplate, 15 mm central post, 36 standard glenosphere, and size 9 x 130 mm** **humeral stem). Both shoulders were stable with good functional ROM. Following irrigation, vancomycin powder and tranexamic acid were placed in the wound. Closure of all layers was done, and dressing was applied. Bilateral shoulder immobilizers were placed. The patient was vitally stable and was extubated and then shifted to the high-dependency unit (HDU) in our hospital for monitoring.

Post-operative hospitalization

**Figure 5:**
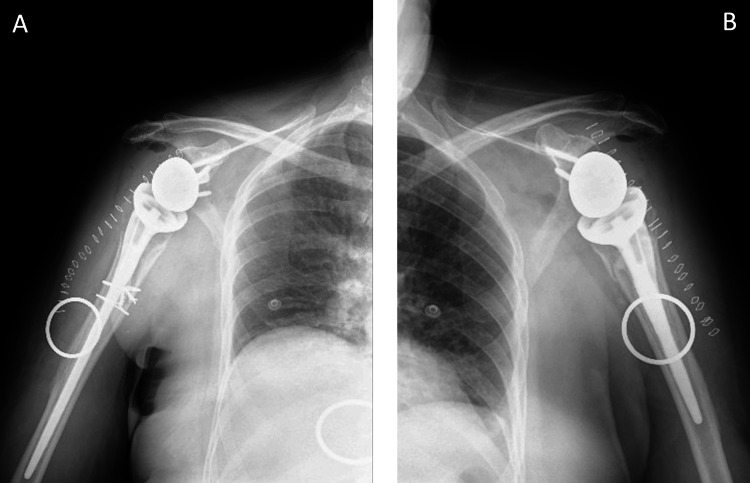
X-ray of the right (A) and left (B) shoulders and humeri (post-operative day 0). (A) Right humerus plain X-ray post-operative anteroposterior view demonstrating reverse total shoulder arthroplasty. (B) Left humerus plain X-ray post-operative anteroposterior view demonstrating reverse total shoulder arthroplasty.

Post-operative X-ray showed good implant positions and good screw length and orientation (Figure 5). However, one day post-surgery, the patient complained of ulnar nerve distribution numbness and pain that was evaluated for. The total blood loss (intra- and post-operative) was 600 mL, and the patient required a transfusion of 1 unit of packed red blood cells. Significant post-operative medications included cefazolin, ciprofloxacin, clexane as a DVT prophylaxis, paracetamol, and intravenous patient-controlled analgesia (IV PCA) for pain management. The patient was discharged five days after the surgery and instructed to perform elbow, wrist, and hand ROM to prevent stiffness. 

Post-operative follow-ups

The arms were kept immobilized for six weeks after surgery with a shoulder immobilizer. The patient was allowed bilateral elbow, wrist, and hand ROM. The patient was seen two weeks after surgery for sutures removal and wound check, which were clean and healed. The patient was started on passive assisted ROM physiotherapy at six weeks post-surgery with gradual progression as per our protocol.

**Figure 6:**
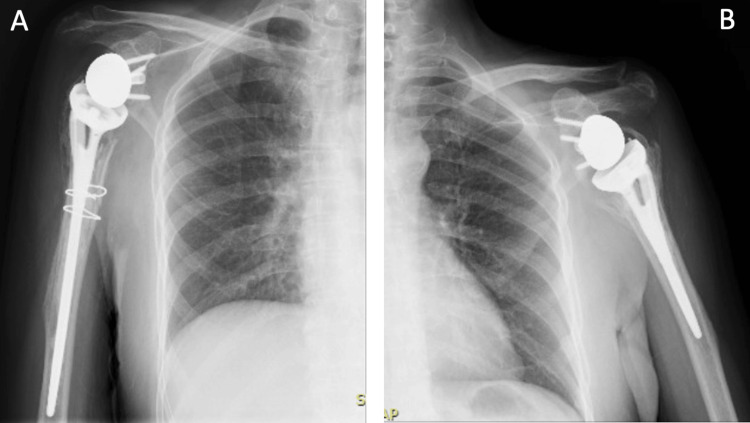
X-ray anteroposterior view of the right (A) and left (B) shoulders and humeri (5 months post-operative) shows no signs of loosening or scapular notching.

**Figure 7:**
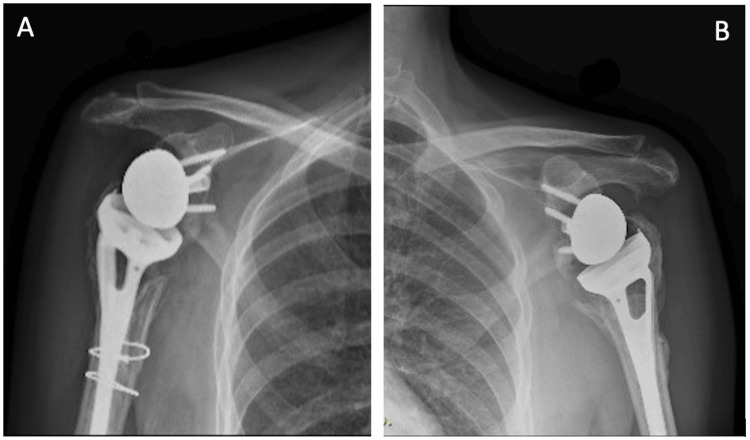
X-ray anteroposterior view of the right (A) and left (B) shoulders (8 months post-operative) shows no signs of loosening or scapular notching.

**Figure 8:**
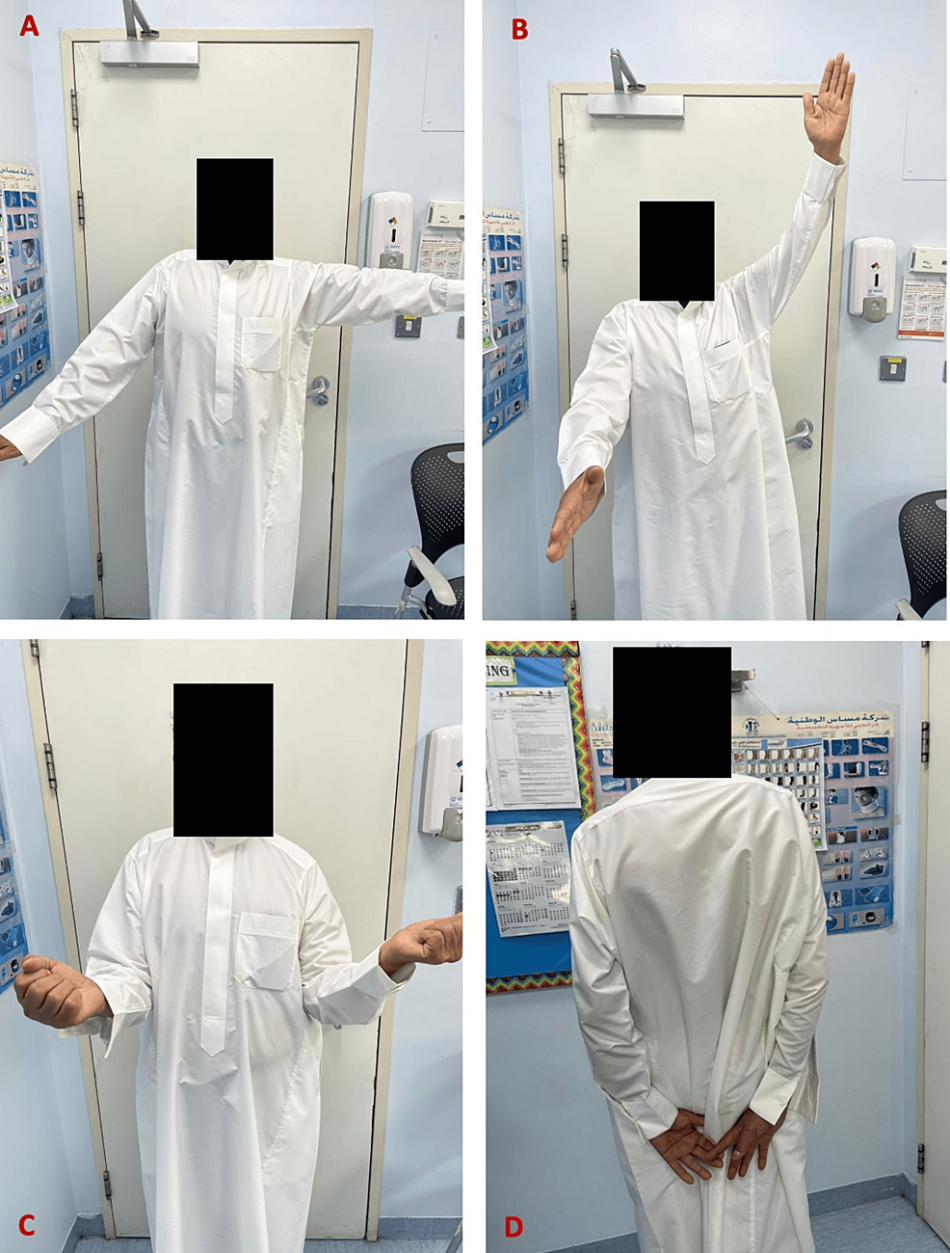
Clinical result at 5-month follow-up: A) Abduction; B) Anterior elevation; C) External rotation; D) Internal rotation.

**Figure 9:**
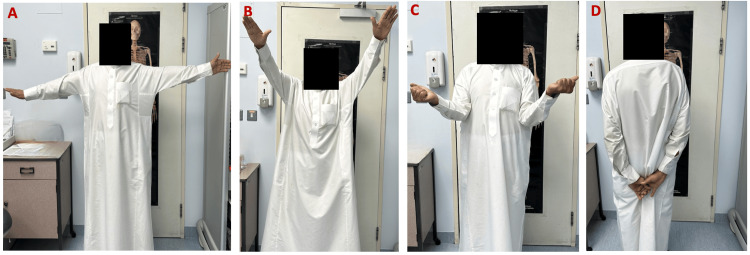
Clinical result at 8-month follow-up: A) Abduction; B) Anterior elevation; C) External rotation; D) Internal rotation.

**Figure 10:**
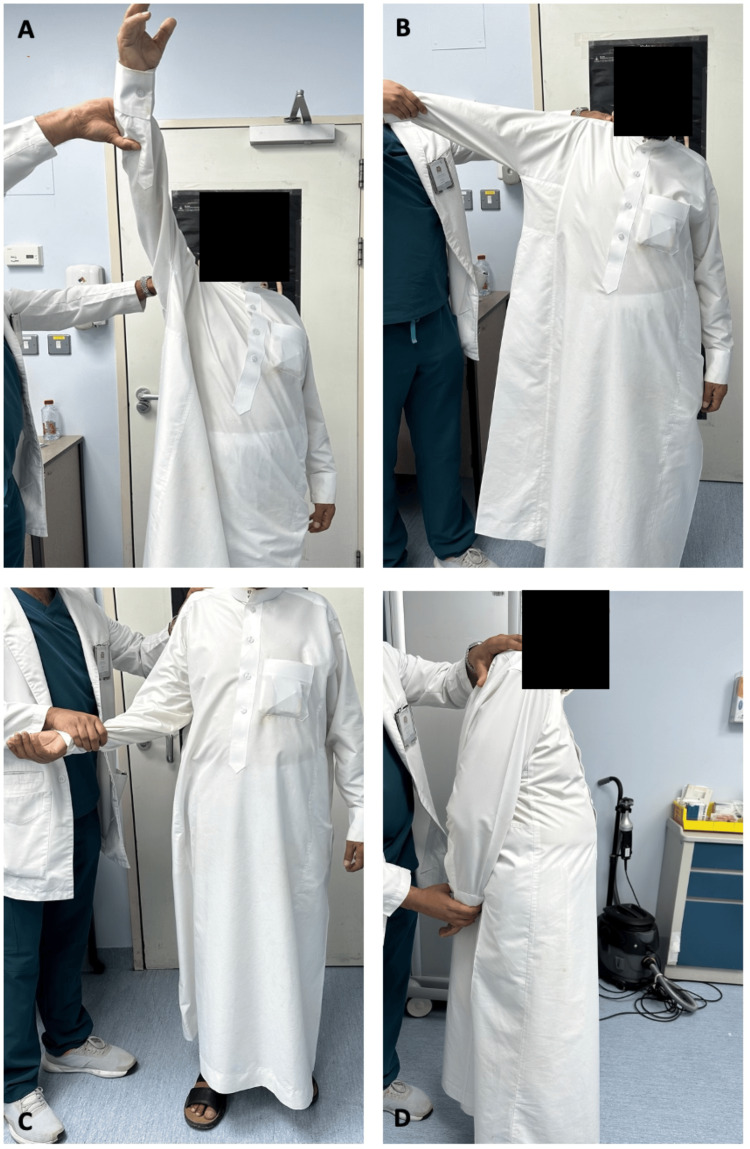
Passive motion of the right shoulder at 8-month follow-up: A) Flexion; B) Abduction; C) External rotation; D) Internal rotation.

Follow-up X-rays were obtained at one, three, five, and eight months from surgery (Figures 6, 7). The latest X-ray revealed good healing and no loosening or scapular notching. Constant-Murley Score (CMS) and the Disabilities of the Arm, Shoulder and Hand (DASH) score were both used for functional assessment at three-, five-, and eight-month follow-ups. In addition, pain was assessed on a numerical rating scale (NRS). Nerve conduction studies confirmed the presence of upper trunk injury of the brachial plexus on the right side which is due to the MVA, otherwise all neurological examinations revealed preserved functions. Table 1 illustrates the approximate NRS, power, ROM, and calculated DASH score and CMS. Figures 8-10 demonstrate the clinical results at the five-month and eight-month follow-ups. 

**Table 1:** The approximate NRS, power, glenohumeral ROM, and calculated DASH score and CMS in follow-up appointments. NRS: Numerical rating scale; ROM: Range of motion; DASH score: Disabilities of the arm, shoulder and hand score; CMS: Constant-Murley score

Follow-up Clinical Results					
3-Month Follow-up					
	Pain (NRS)	Power	Glenohumeral ROM	DASH	CMS
Right shoulder	5	Power of all muscles of upper limb 5/5, except for deltoid muscle 3/5.	Active Forward Flexion: 30^o^, Passive Forward Flexion: 100^o^, Active Abduction: 30^o^, Passive Abduction: 100^o^, External Rotation: 10^o^, Internal Rotation: 20^o^	41	50
Left shoulder	5	Left upper limb power 5/5.	Active Forward Flexion: 90^o^, Passive Forward Flexion: 150^o^, Active Abduction: 90^o^, Passive Abduction: 120^o^, External Rotation: 20^o^, Internal Rotation: 20^o^	14	60
5-Month Follow-up					
	Pain (NRS)	Power	Glenohumeral ROM	DASH	CMS
Right shoulder	3	Power of all muscles of upper limb 5/5, except for deltoid muscle 3/5.	Active Forward Flexion: 40^o^, Passive Forward Flexion: 90^o^, Active Abduction: 40^o^, Passive Abduction: 80^o^, External Rotation: 20^o^, Internal Rotation: 20^o^	38	34
Left shoulder	2	Left upper limb power 5/5.	Active Forward Flexion: 160^o^, Passive Forward Flexion: 160^o^, Active Abduction: 90^o^, Passive Abduction: 110^o^, External Rotation: 35^o^, Internal Rotation: 20^o^	14	66
8-Month Follow-up					
	Pain (NRS)	Power	Glenohumeral ROM	DASH	CMS
Right shoulder	2	Power of all muscles of upper limb 5/5, except for deltoid muscle 4/5.	Active Forward Flexion: 140^o^, Passive Forward Flexion: 160^o^, Active Abduction: 80^o^, Passive Abduction: 90^o^, External Rotation: 25^o^, Internal Rotation: 20^o^	24	40
Left shoulder	1	Left upper limb power 5/5.	Active Forward Flexion: 160^o^, Passive Forward Flexion: 170^o^, Active Abduction: 90^o^, Passive Abduction: 110^o^, External Rotation: 35^o^, Internal Rotation: 20^o^	7.5	68

## Discussion

Based on multiple factors, including patient age, pre-injury activity level, fracture pattern, and surgeon’s experience and preference, non-operative or operative management for PHFs could be determined. Non-displaced or minimally displaced one-part fractures could be treated conservatively with good outcomes. Immobilization is usually the first step in conservative nonoperative management, followed by passive and active range of motion (ROM) and physiotherapy. On the other hand, operative management is indicated in patients with two to four-part PHFs, significant displacement, fracture dislocations, joint instability, or anatomic neck fractures. Surgical options include minimally invasive osteosynthesis, ORIF, closed reduction and percutaneous pinning (CRPP), intramedullary nailing (IMN), hemiarthroplasty (HA), and RTSA. All nerve and vascular injuries, open fractures, and fracture dislocations are considered emergency cases and require immediate referral to orthopedics [4,12,13].

Complexity of the fracture, geometry, the presence of glenohumeral dislocation, AVN, massive rotator cuff tear, and bone quality should all be considered in the pre-operative planning. The selection of RTSA can be attributed to these factors. Distortion of proximal humerus anatomy with complex fractures has been identified as one of the obstacles to tuberosity healing which can result in nonunion or malunion leading to unsatisfactory function. RTSA design depends on a functional deltoid muscle to regain ROM even in the presence of a rotator cuff tear (dysfunctional cuff). Fortunately, RTSA has inherent stability and doesn’t necessarily require subscapularis (lesser tuberosity) healing to provide stability. Compared to hemiarthroplasty, RTSA has improved functional outcomes five years post-operative, with better abduction and forward flexion [16].

In the follow-up of the patient, a plain bilateral shoulder X-ray revealed good healing, with no loosening or scapular notching. A few case reports were published on single-stage or simultaneous bilateral RTSA for bilateral PHFs and reported clinical results that align with our case results. Ceri et al. [6] reported good clinical outcome (CMS and DASH score) and X-ray results at a 1-year follow-up. Azad et al. [7] demonstrated good recovery, function, and ROM of the patient at 4-year follow-up. Iijima et al. [8] showed satisfactory medium-term results in a 4-year follow-up after surgery. El Rassi et al. [9] reported that the combination of RTSA and soft tissue release in a patient with recurrent status epilepticus should reduce failure rate and dislocation. Vitali et al. [10] also demonstrated satisfactory outcomes following one-step bilateral RTSA for posterior fracture dislocation in a young alcoholic. Tokuhiro et al. [5] found that the patient had no restriction of the ADLs 30 months after surgery, but on imaging, bone resorption and superior retraction of the tuberosities were appreciated on both shoulders. 

The orthopedic surgery team justified their decision to proceed with single-stage bilateral RTSA in this particular case because of the complexity and pattern of the fractures, patient factors such as age, pre-injury ADL, and studies showing better outcome after primary RTSA rather than RTSA after osteosynthesis failure in PHFs [17].

## Conclusions

To conclude, short-term follow-up with the patient demonstrated that he was satisfied with the surgery, especially the left side with a pain score on the NRS of 1. Bilateral RTSA is a valid option for the management of bilateral four-part PHF with dislocation. We selected to share our experience of this complex case with our peers in the field so that such a procedure could be implemented in similar cases to ensure satisfactory outcomes following bilateral four-part PHF with dislocation. However, for such a complex condition, we need longer-term follow-ups and a higher level of evidence studies in addition to results from systematic reviews to be able to make robust recommendations.
